# Does Clade Density Constrain Geographical Range Evolution?

**DOI:** 10.1002/ece3.70438

**Published:** 2024-10-31

**Authors:** Marcio R. Pie, Raquel Divieso, Fernanda S. Caron

**Affiliations:** ^1^ Biology Department Edge Hill University Ormskirk Lancashire UK; ^2^ Department of Biodiversity and Conservation Real Jardín Botánico – CSIC Madrid Spain; ^3^ Departamento de Zoologia Universidade Federal do Paraná Curitiba Paraná Brazil

**Keywords:** interspecific competition, range limits, range size evolution, rates of evolution, species distributions, terrestrial vertebrates

## Abstract

The role of biotic interactions, such as interspecific competition, in driving geographical range evolution is still poorly understood. For instance, lineages distributed across regions with a large number of potential competitors might experience some level of geographical packing of their range limits, so that changes in their geographical distributions are hampered. Conversely, a large number of competitors could instead lead to accelerated rates of geographical range evolution, with lineages shifting their ranges to avoid competition. We recently introduced the concept of clade density (CD; the sum of the areas of overlap between a species and other members of its higher taxon, weighted by their phylogenetic distance) as a proxy of the potential for interspecific competition across the geographical distribution of a given species. In this study, we used a large dataset with 5936 terrestrial vertebrate species to test whether CD is significantly associated with variation in the rate of geographical range evolution using two alternative approaches. First, we tested if there is a significant relationship between CD and the geographical distance between sister species. In addition, we estimated tip rates of geographical range evolution and tested if they were consistently associated with variation in CD. We found no evidence for an effect of CD on geographical range evolution in either of the tested approaches, even after accounting for phylogenetic uncertainty. These results are inconsistent with equilibrial models of species diversification and suggest that interspecific competition might not play a pervasive role in geographical range evolution of terrestrial vertebrates.

## Graphical abstract

This study investigates the impact of clade density (CD), a measure of potential interspecific competition, on the geographical range evolution of terrestrial vertebrates. By analysing distribution and phylogenetic data from 5936 species, the results reveal no significant association between CD and either the geographical distance between sister species or the rates of geographical range evolution. These findings challenge the prevailing notion that interspecific competition is a significant driver of range evolution in these taxa.

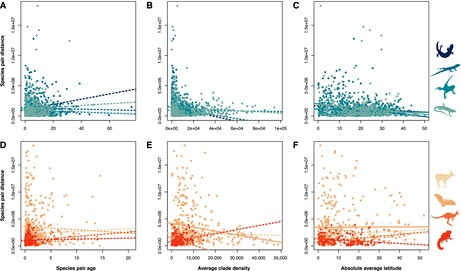

## 1 Introduction

The study of how interspecific competition drives community organisation has a long tradition in ecology (Gause 1934; Hardin 1960; Connell 1961). Indeed, several studies have demonstrated instances of communities being strongly affected by interspecific competition (see Oksanen 1987; Goldberg and Barton 1992; Luiselli 2006). This is particularly true in the case of invasive species, which often lead to severe displacement of native organisms (e.g., Strayer 1999; Martin and Martin 2001). However, even in situations where biotic interactions might be locally strong, that does not necessarily mean that this effect is apparent at broader geographical scales (Prinzing et al. 2002), a phenomenon referred to as Eltonian noise (Soberón and Nakamura 2009; De Araújo, Marcondes‐Machado, and Costa 2014). As a consequence, the extent to which interspecific competition has effects at regional scales is far from obvious, and this theme has received relatively little attention in the literature. One such effect is the potential of interspecific competition to affect geographical range evolution (Price and Kirkpatrick 2009; Henriques‐Silva, Kubisch, and Peres‐Neto 2019). For instance, some studies have argued that there could be competitive release in regions without sympatry between pairs of species, but the evidence for such an effect is far from conclusive (Hansson 1984; Anderson, Peterson, and Gómez‐Laverde 2002; Adams 2010; Neves et al. 2022).

It is intriguing to note that one can envision two alternative and opposite predictions for the potential effect of interspecific competition on the evolution of geographical distributions. For instance, lineages distributed across regions that include a large number of potential competitors might experience some level of geographical packing of their range limits, with lineages arranged “shoulder to shoulder” in geographical space (e.g., Jankowski, Robinson, and Levey 2010; but see Gifford and Kozak 2012). For instance, it has been argued that interspecific competition would limit range expansion after allopatric speciation (Pigot et al. 2010; Pigot and Tobias 2013; Moen and Morlon 2014). This crowding effect could hamper changes in their geographical distributions, whereas species in regions with few competitors would be less constrained to change their range limits. Conversely, one could instead posit an opposite scenario in which a large number of competitors could actually lead to accelerated rates of geographical range evolution, with lineages shifting their ranges to regions with fewer competitors. To the best of our knowledge, neither of those hypotheses has been explicitly tested in datasets that go beyond small groups of closely related species.

Clade density (CD)—the sum of the areas of overlap between a given species and other members of its higher taxon, weighted by their phylogenetic distance—is a concept that has been recently proposed in the context of diversity‐dependent diversification (Pie, Divieso, and Caron 2023). This metric was designed as a proxy for the potential for interspecific competition for two main reasons. First, although sympatry between two species does not necessarily mean that interspecific competition is taking place, they cannot compete with one another if their distribution is allopatric (Salinas‐Ramos et al. 2020). Second, on average, closely related species tend to be similar to one another in a variety of functional traits and therefore are expected to show a higher probability of competing with one another (Richman and Price 1992; Violle et al. 2011; Germain, Weir, and Gilbert 2016). If one assumes that CD is a good proxy for potential interspecific competition, one could expect that there should be a consistent association (positive or negative, see above) between CD and the rate of geographical range evolution.

In this study, we test the prediction that CD influences the evolution of geographical distributions using a large‐scale dataset including 5936 species of terrestrial vertebrates, encompassing a broad range of life histories, habitats, and geographical regions. We used two alternative approaches that predict an influence of CD on the geographic distance between species pairs or on the evolutionary rate of the geographical range. If high interspecific competition hampers the evolution of geographical ranges, we would expect a negative impact of CD on the distance between species pairs or on the evolutionary rate of changes in their geographical range. Conversely, a positive impact of CD would suggest that interspecific competition accelerates geographical range evolution. Our findings reveal no evidence for a consistent effect of CD on geographical range evolution, suggesting that interspecific competition is unlikely to play an important role in governing species distributions at geographical scales, at least in the case of terrestrial vertebrates.

## 2 Methods

### 2.1 Data Collection

We focused on squamates and mammals for this study given that they include two of the largest clades of ectotherm and endotherm vertebrates, respectively, and given the availability of several large‐scale compilations. Phylogenetic relationships for squamates and mammals were obtained from Tonini et al. (2016) and Upham, Esselstyn, and Jetz (2019), respectively. Geographical distributions of species were downloaded from IUCN (2023; version 2023‐1; last accessed on January 30, 2024). The analysis omitted any uncertain ranges classified according to the criteria outlined in the IUCN guidelines (IUCN Standards and Petitions Committee 2024). To expedite computationally intensive calculations, subclades within squamates and mammals were selected for analysis. Also, to facilitate the interpretation of the obtained results, we restricted the analyses to more ecologically homogeneous taxa for which both phylogenetic and distribution data were available, that is, Anguimorpha (*N* = 162), Gekkota (*N* = 1225), Iguania (*N* = 1395), and Scincoidea (*N* = 1216) within squamates, and Cetartiodactyla (*N* = 230), Chiroptera (*N* = 1182), Diprotodontia (*N* = 139), and Primates (*N* = 387) within mammals.

### 2.2 Analyses

We obtained CD for each species as described in Pie, Divieso, and Caron (2023). In brief, we first calculated a range overlap matrix of a given clade, which measures the area of overlap between each pair of species. We then computed the phylogenetic variance–covariance matrix of that clade using their corresponding phylogeny and then calculated the element‐wise multiplication of both matrices. Finally, we sum all of the elements in each line of the resulting matrix to obtain the estimates of clade density for each species (see Pie, Divieso, and Caron 2023 for more details).

We used two alternative approaches to assess the potential influence of CD on geographical range evolution. First, we tested if there is a significant relationship between CD and the geographical distance between sister species. We began by obtaining all sister species pairs in a clade using the function Descendants in *phangorn* v.2.11‐1 (Schliep 2011) and subsetting the sister species. We then calculated the latitude and longitude of the centroid of the geographical distribution of each species using gCentroid function in *rgeos* v.0.6‐4 (Bivand and Rundel 2023) and then used the function distHaversine in *geosphere* v.1.5‐18 (Hijmans 2022) to calculate the geographical distance (in km) between them. Following that, we tested for a relationship between those distances and the mean CD of the corresponding species pair. We included the age of the most recent common ancestor and the absolute average latitude as covariates in the model, given that these variables could potentially influence the geographical distance between sister species. We fit this multivariate regression with a Phylogenetic Generalised Least Squares (PGLS) with the pgls function in *caper* v.1.0‐2 package (Orme et al. 2023).

Second, we estimated tip rates of geographical range evolution and tested if they were significantly associated with variation in CD. We began by estimating the evolutionary tip rates of the midpoint, minimum, and maximum latitude of each species. Two methods were used to calculate these rates, namely the multirateBM function in *phytools* package v.2.3‐0 (Revell 2024) and the RRphylo function in *RRphylo* package v.2.8‐0 (Castiglione et al. 2018). The approaches of the two methods differ in their assumptions about the evolution of the trait. RRphylo is a method based on phylogenetic ridge regression, developed to identify deviations in the rate of evolution in clades or individual species (Castiglione et al. 2018). As this method does not assume any evolutionary model, it cannot misspecify the mode and tempo of trait evolution. On the other hand, multirateBM assumes that the trait evolves under a Brownian Motion, in which the diffusion rate (*σ* ^2^) of the model also evolves under a Brownian Motion but on a logarithmic scale. As a result, both methods make different assumptions about the traits that are important to consider when modelling their evolution. Due to the higher computational cost of the multirateBM function, we had to calculate the tip rates from subtrees consisting of 50 species at most each time. Therefore, the phylogenies of each taxon were subdivided into subtrees prior to the calculations with this method. Preliminary analyses showed that this approach provides a good approximation for the rates calculated using the whole phylogeny (not shown) while also making it possible to run the analyses in a reasonable time. For the tip rates calculated using the RRphylo function, we were able to use the entire phylogenies, including the variable being assessed as a covariate in the function, as recommended by the authors (Castiglione et al. 2018). We adopted this approach because RRphylo tends to estimate rates that show a correlation with the trait value (Castiglione et al. 2018). For every set of tip rates of each latitude metric, we performed a PGLS analysis using the species CD as a predictor, given that there is high phylogenetic signal on CD across all tested clades (Table S1). We repeated all analyses for 100 alternative topologies to account for phylogenetic uncertainty. All analyses were performed in R 4.2.2 (R Core Team 2023).

## 3 Results

**FIGURE 1:**
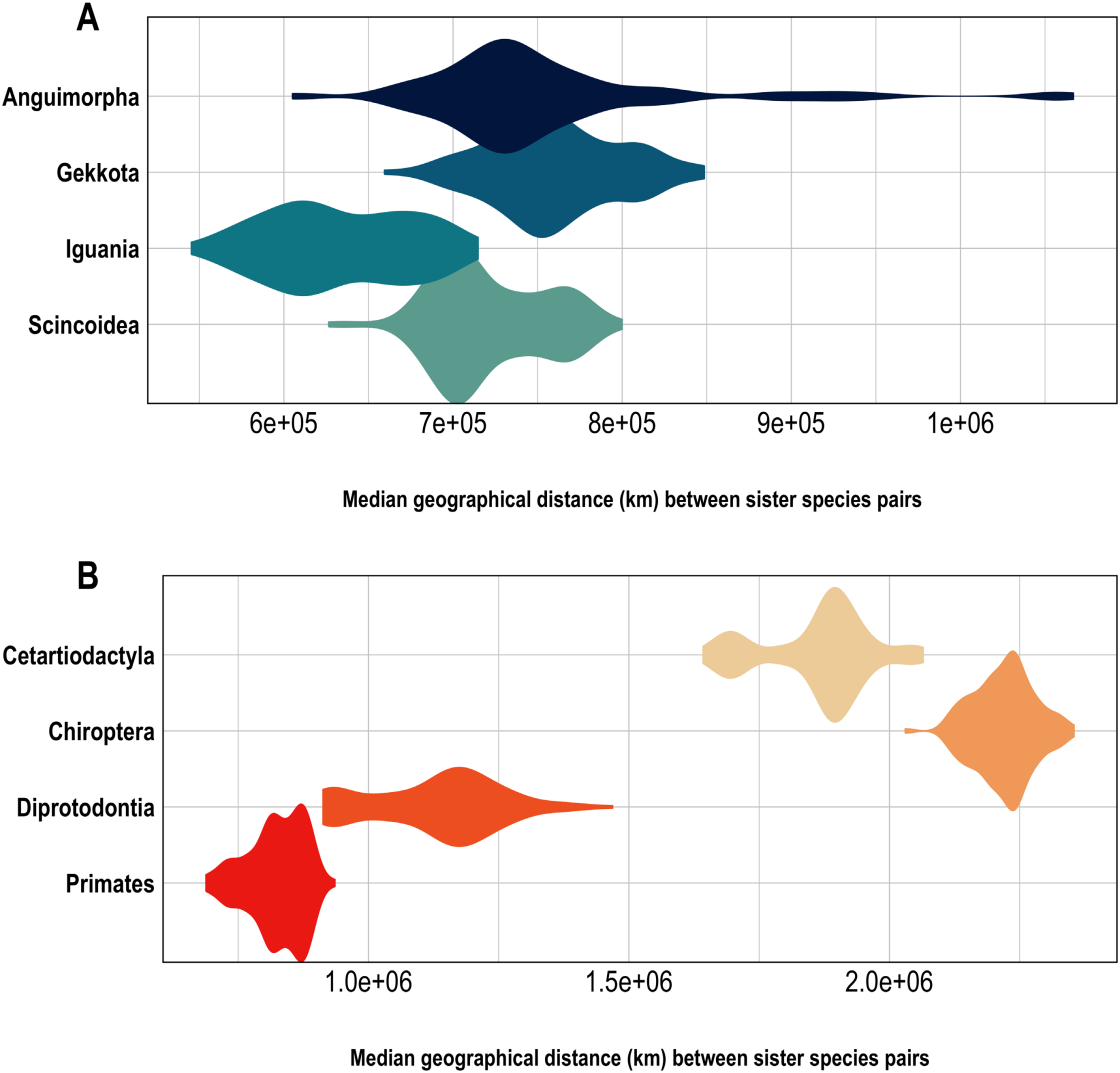
Distribution of the median geographical distance in km between sister species pairs for squamates (A) and mammals (B) across the 100 trees used in the analyses.

There were substantial disparities across taxa in the geographic distances separating sister species. The median geographical distances for species pairs across the 100 trees were notably smaller within all orders of squamates (Figure 1A) when compared to mammals’ orders (Figure 1B). Among all examined trees, the lowest average geographical distance between species pairs of squamates was found for Iguania (633,270.5 km), whereas for mammals, Primates exhibited the lowest average distance between species pairs (824,953.4 km), whereas the largest average distances were observed between Gekkota (760,450.5 km) and Chiroptera (2219,458.2 km), for squamates and mammals, respectively. The medians, standard deviations, minimum and maximum values of geographical distances between species pairs across each of the 100 utilised trees are presented in Table S2.

**FIGURE 2:**
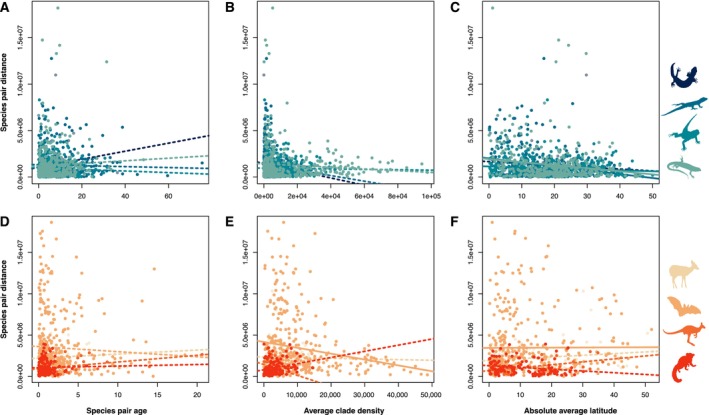
Relationships between species pairs’ age, average clade density, and absolute latitude and species pairs distance. (A, D) Relationships between species pair age and geographical distance. (B, E) Correlations between species pair average clade density and their geographical distance. (C, F) Relationships between the species pair absolute average latitude and their geographical distance. The top row corresponds to squamates (silhouettes from top‐down: Anguimorpha, Gekkota, Iguania, and Scincoidea) and the bottom row to mammals (Cetartiodactyla, Chiroptera, Diprotodontia, Primates). Lines represent the tested regressions for one topology, with solid lines corresponding to the significant relationships and shaded lines to non‐significant ones.

**FIGURE 3:**
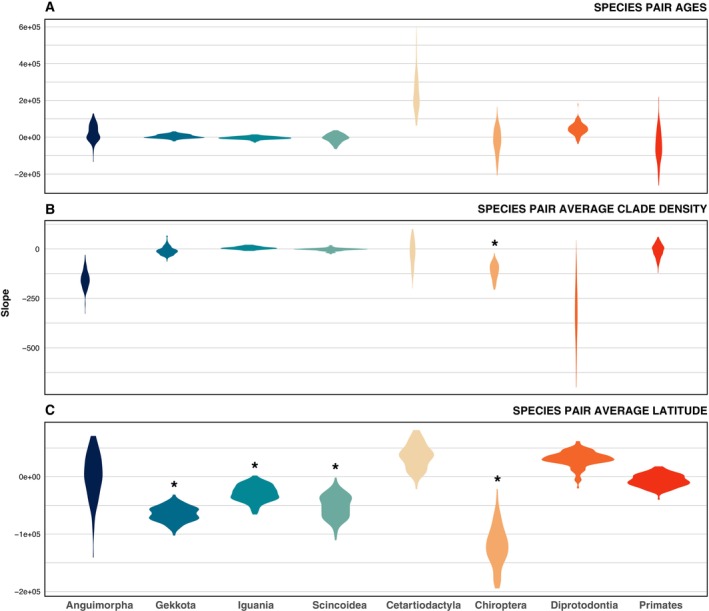
Slopes of the tested relationships between species pairs' age, average clade density, and absolute latitude and species pairs' distance. (A) Slopes for the relationship between species pair age and geographical distance. (B) Slopes for correlations between species pair average clade density and their geographical distance. (C) Slopes for the relationship between the species pair absolute average latitude and their geographical distance. The range of values represented corresponds to the phylogenetic uncertainty. Asterisks correspond to statistically significant relations.

As our first approach to assess the potential effect of CD on geographical range evolution, we tested the correlation between CD and the geographical distance between species pairs while including species pairs' age and average latitude as covariates. Overall, our results did not reveal consistent evidence of correlations between CD and geographical range evolution across the analysed taxa (Figures 2 and 3; Table S3). More specifically, the majority of taxa did not show evidence for an effect of average species pair CD on their geographical distance, except for Chiroptera (Figures 2B,E and 3B; Table S3). However, this exception can be explained by the multiple tests performed in our study, and would not be significant if any *p*‐value correction was employed. Similarly, species pair age did not present a significant effect on species pair geographic distance for any taxa (Figures 2A,D and 3A; Table S3). In contrast, average species pair latitude was generally negatively related to the species pair geographic distance in Gekkota, Iguania, Scincoidea, and Chiroptera (Figure 3C; Table S3). In other words, for these groups, sister species located closer to the tropics tend to exhibit greater geographical proximity between them. Taking phylogenetic uncertainty into account did not change our conclusions (Table S3).

**FIGURE 4:**
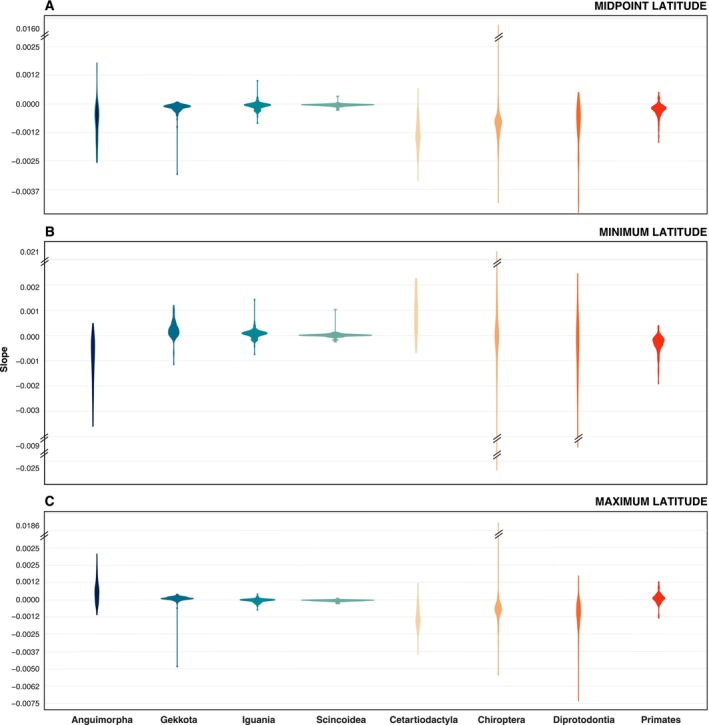
Slopes of the tested relationships between species clade density, and the evolutionary rate of geographic range metrics. Relationships are presented between clade density and the evolutionary rate of midpoint (A), minimum (B), and maximum latitude (C). The range of values represented corresponds to the phylogenetic uncertainty. Asterisks correspond to statistically significant relations (none are shown).

In our second approach for assessing how CD might affect the evolution of geographical ranges, we looked at how the rates of evolutionary change at the midpoint, minimum, and maximum latitude of each species range relate to the variation in CD. Again, our results obtained did not show statistical significance for any taxon (Figure 4; Tables S3 and S4). Notably, this lack of correlation was consistent across all range metrics, that is, midpoint, minimum, and maximum latitude (Figure 4). Even when employing alternative methods to estimate evolutionary rates, the regimes of evolutionary change in none of the traits related to geographic distribution appear to be influenced by their respective CD (Table S5). These results are also robust to phylogenetic uncertainty (Tables S4 and S5).

## 4 Discussion

In this study, we tested the hypothesis that rates of geographical range evolution would change consistently according to variation in clade density (CD) using a large‐scale dataset of terrestrial vertebrates. We found no evidence for such an association for either of the tested approaches, even after accounting for phylogenetic uncertainty. It is important to note that the studied taxa include a broad range of life histories, dispersal capacities, habitats, and geographical regions, providing ample opportunity for a potential effect of CD on range size evolution to be detected, if present. These results indicate that interspecific competition is unlikely to play a major role as a driver of geographical range evolution. On the other hand, our results corroborate the observation that sister species tend to be younger in tropical regions, as previously shown for birds and mammals (Weir and Schluter 2007).

Although there are many examples of interspecific competition as an important driver of community organisation (e.g., Baron et al. 2015), its relevance might have been overestimated in macroevolutionary studies. For instance, interspecific competition does not seem to be a major organising force in many communities, and even where it can be demonstrated, it need not have a major role in community structuring (e.g., Shorrocks et al. 1984; Wellings 1987). Indeed, other mechanisms seem to counteract the impact of interspecific competition, such as intraspecific aggregation (Stoll and Prati 2001), intraspecific competition (Martorell and Freckleton 2014; Adler et al. 2018), and stochasticity (Edwards and Stachowicz 2011). The lack of correlation between clade density and species geographical range evolution, as well as their speciation rates (Pie, Divieso, and Caron 2023), raises doubts about whether interspecific competition consistently imprints discernible patterns on large‐scale macroevolutionary processes (see also Harmon and Harrison 2015). It is important to note that diversification slowdowns, which are often assumed to reflect diversity‐dependent diversification, are not only far from prevalent, but also can be explained by mechanisms other than competition (Moen and Morlon 2014).

It is important to note some relevant caveats about our analyses. First, variation in taxonomic traditions (the traditional dichotomy between “splitters” and “lumpers”) has been shown as a potential bias in diversification studies (Faurby, Eiserhardt, and Svenning 2016). Indeed, taxonomic overdescription is likely to be most pronounced in species‐rich groups, where many species have relatively small geographic range sizes and have low numerical abundance (Jones, Purvis, and Quicke 2012). This could be particularly problematic at low latitudes, given that they tend to be areas of high diversity and relatively fewer trained taxonomists (although in this case there could be fewer descriptions from splits of previously known species, given there could potentially be fewer taxonomic reviews). Our analyses cannot rule out those effects, and at the moment we cannot estimate empirically their impact. However, we believe that, even if present, these potential inaccuracies in taxonomic assignments are unlikely to fundamentally change our conclusions for three main reasons. First, given that terrestrial vertebrates tend to be among the best‐known taxa with respect to their distribution and taxonomy, when compared to most other animal taxa, their species have been under considerable scrutiny for nearly three centuries. Second, our analyses included a broad sample of animal taxa with a variety of ecologies, life histories, and geographical distributions. Therefore, if there was an important impact of CD on geographical range evolution, we should have detected consistent associations in at least some of the studied clades, which we did not. Third, it is unlikely that widely distributed, terrestrial vertebrate species are still undescribed, and the still undiscovered species are likely to come from particular habitats in relatively small ranges (e.g., islands), which by definition would not exert an important CD effect on its closely related lineages. Fourth, our conclusions depend fundamentally on the validity of our data on species geographical distributions. There are two main ways in which they could affect our inferences. Geographical distributions could be so labile to the point that any effects at deeper evolutionary timescales could be masked. Although there are several examples of relatively fast changes in distributions in many species, the vast majority of studies on the phylogenetic signal of geographical distributions found strong evidence for phylogenetic autocorrelation, both in size and geographical position (e.g., Waldron 2007; Pie and Meyer 2017; Pie et al.  2021). These results suggest that, even though geographical distributions do change, they typically tend to evolve at a rate that should still reflect the effect of interspecific interactions on geographical ranges in the way envisioned in our analyses, if present. Alternatively, there could be errors in current estimates of species distributions. Although there is an inherent level of uncertainty when dealing with such vast geographical and taxonomic scales, the IUCN database has been continuously improved by experts and is likely to represent fairly reasonable approximations, which indeed provide similar results when compared to alternatives such as georeferenced occurrence records (e.g., Alhajeri and Fourcade 2019). Therefore, despite these caveats, we believe that our analyses are robust and should have been able to detect an effect of CD on geographical range evolution, if present.

Recent studies have demonstrated predictable patterns related to geographical range evolution. For instance, cold limits (geographical limits closer to the poles) tend to evolve substantially faster than warm limits (those closer to the equator) (Pie and Meyer 2017; Pie et al. 2021), and geographical range evolution tends to be faster in endotherms (Pie, Divieso, and Caron 2021). The discovery of these striking patterns in recent years suggests that there are indeed general principles governing geographical range evolution. However, they also underscore the influence of other variables on geographical range evolution beyond local interactions. Indeed, it appears that biotic interactions either become less influential at larger spatial scales (HilleRisLambers et al. 2012) or that multiple simultaneous factors obscure the impact of interspecific competition.

## Author Contributions

**Marcio R. Pie:** conceptualization (lead), funding acquisition (lead), methodology (lead), project administration (lead), supervision (lead), writing – original draft (lead), writing – review and editing (lead). **Raquel Divieso:** data curation (equal), formal analysis (equal), visualization (equal), writing – original draft (supporting), writing – review and editing (equal). **Fernanda S. Caron:** data curation (equal), formal analysis (equal), visualization (equal), writing – original draft (supporting), writing – review and editing (equal).

## Conflicts of Interest

The authors declare no conflicts of interest.

## Supporting information


**Table S1.** Tests of phylogenetic signal of clade density estimates, using Pagel’s *λ*. All tests were carried out using the phylosig function in *phytools* v. 2.3‐0 (Revell 2024). The value refers to the average value across topologies, with 95% confidence intervals enclosed in parenthesis.

**Table S2.** Summary statistics of geographical distances (km) between species pairs, calculated for each clade across 100 phylogenetic trees.

**Table S3.** Phylogenetic generalised least squared analyses of the relationship between species pair distances and species ages, clade density, and absolute latitude. The value refers to the average value across topologies, with 95% confidence intervals enclosed in parenthesis.

**Table S4.** Phylogenetic generalised least squared analyses of the relationship between tip rates and midpoint, minimum, or maximum latitude. Tip rates were calculated with the multirateBM function (see Section 2). The value refers to the average value across topologies, with 95% confidence intervals enclosed in parenthesis.

**Table S5.** Phylogenetic generalised least squared analyses of the relationship between tip rates and midpoint, minimum, or maximum latitude. Tip rates were calculated with the RRphylo function (see Section 2). The value refers to the average value across topologies, with 95% confidence intervals enclosed in parenthesis.

## Data Availability

All data used in this study are sourced from the public domain. Detailed information on the respective repositories can be found in Section 2.1.
